# Citrus Segment Degradation Potential, Enzyme Safety Evaluation, and Whole Genome Sequence of *Aspergillus aculeatus* Strain ZC-1005

**DOI:** 10.3389/fmicb.2021.671200

**Published:** 2021-08-20

**Authors:** Jieyi Wang, Zhipeng Gao, Yujiao Qian, Xiao Hu, Gaoyang Li, Fuhua Fu, Jiajing Guo, Yang Shan

**Affiliations:** ^1^Hunan Agriculture Product Processing Institute, International Joint Laboratory on Fruits and Vegetables Processing, Quality and Safety, Hunan Key Laboratory of Fruits and Vegetables Storage, Processing, Quality and Safety, Hunan Academy of Agricultural Sciences, Changsha, China; ^2^College of Animal Science and Technology, Hunan Agricultural University, Changsha, China; ^3^Longping Branch, Graduate School of Hunan University, Changsha, China

**Keywords:** *Aspergillus aculeatus* ZC-1005, degradation, safety evaluation, genome sequencing, xylanase

## Abstract

*Aspergillus aculeatus* ZC-1005 (ZC-1005 was used as the abbreviation of this strain) is a hemicellulase-producing strain isolated from rotten citrus rind buried in the soil. Our previous study has shown its biochemical properties including high xylanase activity, mannanase activity, and degradation reaction with citrus mesocarp. In this study, we focused more on the enzyme safety evaluation and the genome sequencing *via* PacBio and Illumina platforms. High biological safety of the crude enzymes of ZC-1005 has been proven by the acute oral toxicity test, sub-chronic toxicity test, micronucleus test, and sperm malformation test. The genome of ZC-1005 had a GC content of 52.53%, with a size of 35,458,484 bp, and encoded 10,147 genes. Strain ZC-1005 harbored 269 glycosyl hydrolase (GH) genes of 64 families. The fungus produces cellulose-acting (GH3, GH5, GH12, and GH1) and hemicellulose-acting enzymes (GH16, GH31, GH2, and GH92). In genome annotation, we paid more attention to the genes encoding xylanase, such as gene 01512, gene 05833, gene 05469, gene 07781, gene 08432, gene 09042, gene 08008, and gene 09694. The collaboration between complete genome information and the degradation test confirmed that ZC-1005 could degrade cellulose and xylan. Our results showed that the citrus enzymatic decapsulation technology was efficacious and safe for canned citrus product processing, which may also solve the industrial waste problem. Therefore, ZC-1005 and the crude enzyme secreted from the strain were very promising to be used in the citrus processing industry.

## Introduction

China is a major producer of citrus canned products (Miao et al., 2013; Shan, 2013). Decapsulation technologies determine the quality of citrus products, which are crucial for the whole production process (Shan et al., 2009). The cutting-edge enzymatic stripping technology has significantly improved the quality of the citrus canned products, causing less environmental pollution (Zhang et al., 2008). At present, pectinase and cellulase are the main enzymes used in enzymatic stripping (Long et al., 2013), while the enzymatic targeting on the hemicellulose component was underemphasized.

Citrus fruits are composed of an outer covering cortex and pulp containing juice. Each orange flap is wrapped by a thin film, a sac tissue, commonly known as citrus mesocarp. The degradation of citrus mesocarp is an important step in citrus processing. At present, the main methods used to degrade mesocarp are the classical acid–base two-step method, phosphate one-alkali method, EDTA-assisted low alkali method, and enzymatic decapsulation technology (Li et al., 2013). Compared with other methods, enzymatic decapsulation technology has become a hot spot because it is a type of biological method. This method decomposes pectin, cellulose, and hemicellulose in the citrus capsule by enzyme but not destroying the pulp (Shan et al., 2009). The citrus enzymatic decapsulation technology was established by our research group and first applied in Hunan Fruitops Ltd., Co., (Yongzhou, China). The technology solved the industrial waste problem and improved the product quality and safety (Wang and Jiang, 2011).

The chemical composition of the mesocarp mainly includes pectin, cellulose, hemicellulose, and other substances (Shang, 2004). In our previous study, we isolated a fungus of ZC-1005 that showed lignocellulose-degrading ability (Zhang, 2014). Lignocellulose mainly consists of cellulose, hemicellulose, and lignin. Hemicellulose is a kind of polysaccharide in the plant cell wall, which is located below lignin and cellulose. With the help of hemicellulose, lignin and cellulose tightly bond with each other (Saha, 2003). Cellulose, hemicellulose, and lignin establish the supporting system of plant cell wall (Fischer and Bennett, 1991). Hemicellulose is a heteromultimer composed of several different types of monosaccharides, such as D-xylose, D-mannose, D-arabinose, and D-galactose (Haiping et al., 2007; Scheller and Ulvskov, 2010). Xylan, the main component of hemicellulose, is a kind of complex polypentacarbon sugar. The main chain is formed by β-D-pyrazinyl xylose residues polymerized by β-D-1,4 xylose bonds. Xylan can be partially degraded to form xylooligosaccharide, and thoroughly degraded into pentacarbon monosaccharides, including xylose, ferulose, arabinose, etc, among which xylose is the main component. Its degradation enzymes mainly include endo-β-xylanase (acting on the main chain) (Khandeparker and Numan, 2008), exo-xylanase (acting on oligosaccharides and non-reducing ends of xylan) (Ahmed et al., 2017), and other enzymes like α-D-glucuronidase and α-L-arabinofuranosidase (acting on the branch chain of xylan) (Tony et al., 2010).

To explore the potential applications of this strain, the whole genome sequencing of this strain was performed. Citrus mesocarp degradation experiments, enzyme activity measurements, and animal tests were also conducted. The findings of this study would benefit the development of gene engineering and enzyme engineering, and the industrialization of enzyme production.

## Materials and Methods

### Fungal Strain

The fungal strain used in this study was *Aspergillus aculeatus* ZC-1005 (CCTCC NO: M 2013324) isolated from the rotten oranges buried in soils in our previous studies and kept in the China Center for Type Culture Collection (CCTCC) (Zhang, 2014). The strain was cultured in PDA medium at 35°C.

### The Production of Crude Enzymes

To induce the secretion of xylanase enzymes of ZC-1005, bran and Czapek salt solution were used as carbon source and nitrogen source, respectively. The method used was described in previous studies (Zhang, 2014). The spores of ZC-1005 were washed with 0.9% NaCl to make a spore suspension (1 × 10^6^/ml) and then 1 ml of the suspension was transferred into an Erlenmeyer flask with 75 ml of fresh medium and incubated aerobically at 35°C with shaking at 170 r/min for 60 h; after incubation, the fungal suspension was filtered with gauze and the filtrates were centrifuged (8,000 *g*, 4°C, 10 min) to separate the supernatant; finally, the supernatant was filtered through a 0.22-μm filter membrane (Millipore, United States) and the filtrates were collected as the crude enzymes used in this study.

### The Degradation of Citrus Mesocarp

The citrus segments with uniform mass and size were collected and then the segments were degraded by the crude enzyme solution [ratio: segments/crude enzyme solution (W/W), 1:5] in a water bath at 50°C; the mixed solution was taken out every 10 min and gently shaken 5–10 times. During this degrading process, the images of the degraded mesocarp were taken at 10, 20, 30, 40, and 50 min, respectively. At the same time points, the paraffin sections of mesocarp were also prepared and dyed by toluidine blue for 2–5 min; after washing three times with PBS, the slices were dried and sealed with neutral gum. Finally, the slices were observed using an upright optical microscope (NIKON ECLIPSE E100, Japan) and the images were collected.

### The Evaluation of the Toxicity of Crude Enzymes

Generally, determining the biosafety of the crude enzymes was carried out based on the National Standard of the People’s Republic of China: Procedures for Toxicological Assessment on Food Safety (The Ministry of Health of the People’s Republic of China, 2014).

#### The Feeding Environment

Subjects were placed in a stable environment (temperature 19–25°C, relative humidity 40–50%, 12-h light–dark cycle) and adapted to this environment for 1 week before used. All the animals (ICR mice and SD rats) used in this study were provided by Hunan Silaike Jingda Co. (Changsha, China). The study was reviewed and approved by the Institutional Animal Care and Use Committee (IACUC) of Hunan Silaike Jingda Co., with certificate no. IACUC-SJA18072. All animal protocols were in accordance with the relevant Regulations of The People’s Republic of China on laboratory animal welfare and the Regulation on Management of Experimental Animals (Hunan Province, No. 259, 2012).

#### Acute Oral Toxicity Test

The maximum tolerated dose (MTD) method was taken in the acute oral toxicity test. A total of 40 ICR mice (18–22 g, 20 males and 20 females) were randomized to receive sterile distilled water (group 1: control group, *n* = 20) or crude enzyme solution (group 2: treated group, *n* = 20). After fasting for 12 h, 40 ml/kg of the crude enzyme solution and an equal volume of sterile distilled water were orally administered to the treated group and control group, respectively.

After administration, the mice were kept under observation for 0–4 h, lasting for 14 days. The daily observations of the mice included appearance, behavioral activities, secretions, excretions, diet, and death (time of death and pre-death reaction). The body weight of each mouse was recorded on days 0, 7, 10, and 14 of administration. After 14 days of administration, the mice were dissected and the macroscopic necropsy was carried out, including inspection for the outer surface of the body and the morphological characteristics of the major organs (liver, kidney, spleen, heart, and brain).

#### Sub-Chronic Toxicity Test

Twenty Sprague–Dawley (SD) rats (4 weeks old; 10 males and 10 females) were subjected to sub-chronic toxicity test. The rats were divided into two groups: 10 rats in each group were individually caged. During the 28-day experiment, 10 ml/kg of the crude enzyme solution and an equal volume of sterile distilled water were orally administered to the treated group and control group daily. The general performance, behavior, poisoning, and the death of the rats were observed and recorded daily. The rats were weighed once a week. The food conversion efficiency was calculated every week.

After 28 days of administration, the blood samples were collected from all the surviving rats for blood analysis. Firstly, the platelet count (PLT), hemoglobin concentration (Hb), white blood cell count (WBC) and classification, and red blood cell count (RBC) were measured by Full-Automatic Blood Analyzer DF50 (Shenzhen Dymind Biotechnology Co., Ltd.). Then, the serum was collected by centrifuging. Serum glucose concentration, cholesterol, triglycerides, aspartate aminotransferase (AST), alanine aminotransferase (ALT), creatinine, serum albumin, total protein, and bilirubin were measured by Automatic Biochemical Analyzer FAITH-1000 (Nanjing Laola Co., Ltd., China). Finally, the rats were decapitated and the organs (kidneys, liver, spleen, and testicles) were weighed.

#### Micronucleus Test

A total of 50 ICR mice (weighing 18–25 g; 25 males and 25 females) were used in micronucleus test. The mice were equally divided into five groups: low-dose enzyme solution group, medium-dose enzyme solution group, high-dose enzyme solution group, negative control group, and positive control group. The high-dose enzyme solution group received enzyme solution of MTD, and the other two groups received enzyme solution of 50% and 25% of MTD, respectively. Sterile distilled water was administered to the negative control group, and the positive control group was treated with 40 mg/kg cyclophosphamide. The same administration was repeated again 24 h later. Finally, the mice were sacrificed by cervical dislocation and the bone marrow smears were prepared 6 h later by using fetal calf serum.

Cell counting was performed by a hematocytometer. Under high-power magnification (10 × 100), micronuclei were detected in polychromatic erythrocytes (1,000 per animal) under oil immersion. To estimate whether bone marrow cell division was inhibited, the polychromatic erythrocytes (PCE)/normochromic erythrocytes (NCE) ratio was calculated for 1,000 cells.

#### Sperm Malformation Test

Thirty male ICR mice were equally divided into five groups (six mice per group): low-dose enzyme solution group, medium-dose enzyme solution group, high-dose enzyme solution group, negative control group, and positive control group. The high-dose enzyme solution group received enzyme solution of the maximum dose, and the other two groups received 50% and 25% of the original solution, respectively. The negative control group was given distilled water by gavage and the positive control group was given cyclophosphamide 40 mg/kg once a day for five consecutive days. After 35 days of administration, all the mice were sacrificed and the epididymal sperm suspension was prepared at 37°C and then the sperm smears were subsequently prepared and stained with eosin. Finally, the smears were observed by a light microscope and the sperm aberration rate of 1,000 sperm cells per mouse was calculated.

### Sample Preparation and Genome DNA Extraction

The genomic DNA of ZC-1005 was extracted by using the Omega Fungal DNA Kit D3390-02 (OMEGA Bio-tech, United States) following the manufacturer’s protocol (Majorbio Company, Shanghai, China). The purified genomic DNA was quantified by a TBS-380 fluorometer (Turner BioSystems Inc., United States). High-quality DNA (OD_260/280_ = 1.8–2.0, > 15 μg) was used for further research. The extracted genomic DNA was analyzed by a NanoDrop 2500 spectrophotometer (Thermo Fisher, United States) to check its purity (A_260/280_ ratio) and concentration.

### Library Construction and Sequencing

PacBio Sequel Single Molecule Real-Time (SMRT) and Illumina sequencing platforms were used for genome sequencing. The complexity of the genome was assessed according to Illumina data. At least 5 μg of genomic DNA was required to construct a sequencing library for Illumina sequencing. DNA samples were cut into 400- to 500-bp fragments by using the Covaris M220 Focused Acoustic Shearer. The Illumina sequencing library was prepared from the cut fragments with the NEXTflex^TM^ Rapid DNA-Seq Kit. Simply put, the 5′ prime ends were the first end-repaired and phosphorylated. Next, the 3′ ends were A-tailed and connected to sequencing adapters. The third step is to use PCR technology to enrich adapter-ligated products. The paired-end Illumina sequencing (2 bp × 150 bp) was then performed on the Illumina HiSeq X-Ten machine using the prepared libraries.

For Pacific Biosciences sequencing, in a Covaris g-TUBE (Covaris, MA, United States), an Eppendorf 5424 centrifuge (Eppendorf, NY, United States) was used to rotate 8-μg DNA aliquots at 6,000 rpm for 60 s. SMRTbell sequencing adapters were used to purify, end-repair, and ligate the DNA fragments. The purification for the obtained sequencing library was repeated three times with Beckman Coulter genomics (MA) of 0.45 times volume as instructed by the manufacture. Next, a ∼10-kb insert library was prepared and sequenced on a SMRT cell.

### Assembly and Annotation

Bioinformatics analysis was performed using data generated by the Pacbio and Illumina platforms. I-Sanger Cloud Platform^1^ from Shanghai Majorbio was used to conduct all the analyses. The detailed procedures are as follows.

#### Genome Assembly

PacBio reads and Illumina reads were used to assemble the genome sequence. The genome sequence was a combination of PacBio and Illumina reading codes. The sequence data, defined as raw data or raw reads, involved the conversion of the original image data through base calling and were saved as FASTQ files. These FASTQ files are raw data provided to users, including read order and quality information. The method of quality information statistics was used for quality trimming, removing low-quality data, and forming clean data. The reads were then assembled into contigs using CANU. Finally, Illumina reads were used to correct the error of Pacbio assembly results.

#### Genome Annotation

Maker2 was used for the identification for the predicated coding sequence (CDS), also known as open reading frames (ORFs), tRNA-scan-SE for tRNA prediction, and Barmap for rRNA prediction. Genome functional annotation was based on the BLASTP and Kyoto Encyclopedia of Genes and Genomes (KEGG), Clusters of Orthologous Groups (COG), Gene Ontology (GO), the non-redundant Protein (NR)/Swiss-prot, Pfam, and Carbohydrate-Active enzymes (CAZy) database. In short, each set of query proteins was aligned with the database, and the best matched subject’s annotation (E-value < 10^–5^) was obtained for gene annotation.

### Statistical Analysis

Statistical analysis was performed by SPSS software 16.0. The results were expressed as mean ± SEM. *P* values less than 0.05 were considered statistically significant.

## Results

### Degradation Effect of the Crude Enzyme Solution on Citrus Segments

**FIGURE 1:**
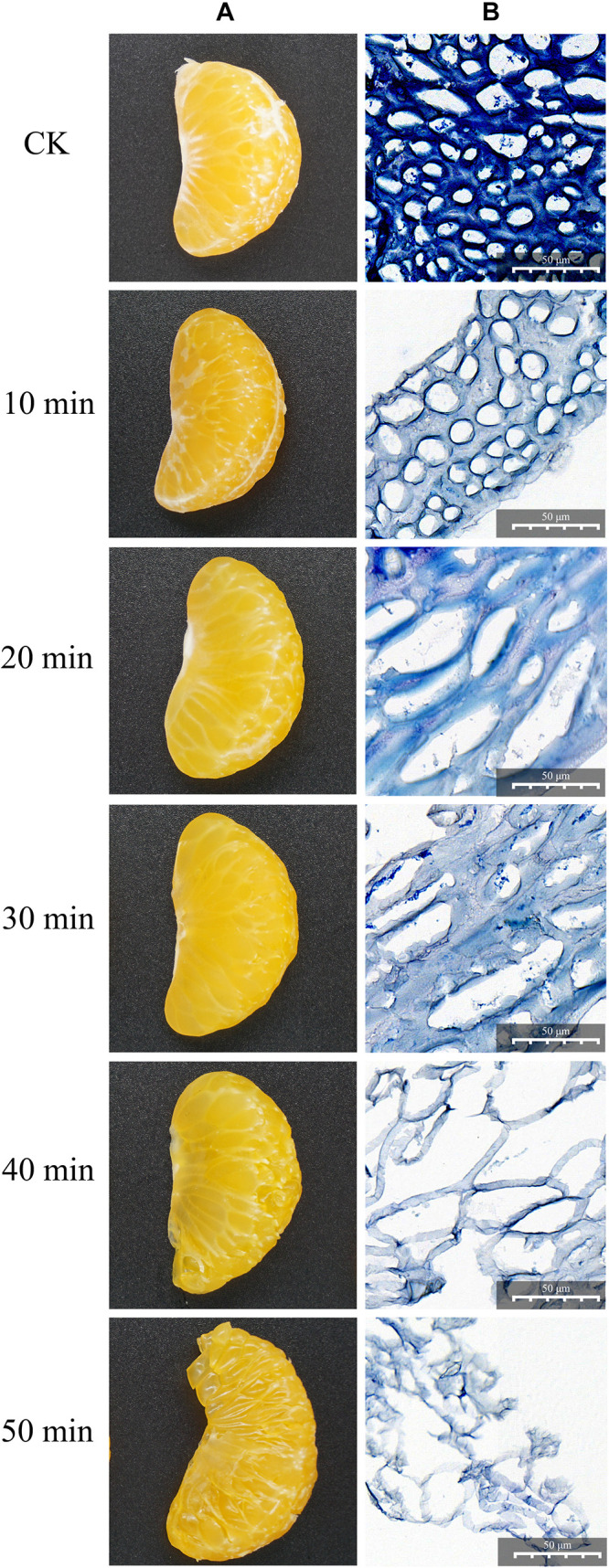
Degradation effect of the crude enzyme solution on citrus segments at different time points. **(A)** Macroscopic observation. **(B)** Microscopic observation by light microscopy.

As shown in Figure 1, the degradation effect was different at different enzymolysis times. Through macroscopic observation (Figure 1A), the fresh citrus segments were degraded gradually in a time-dependent manner; the segments were degraded completely after 50 min of treatment. In order to see more details, the citrus mesocarps were collected, dyed in blue, and observed in a light microscope (Figure 1B). Without any treatment, the citrus mesocarp was composed of 10–20 layers of parenchyma cells; the inner layer was composed of large cells with an irregular arrangement, while the surface cells were small, arranged orderly and tightly, and there were cellulose and pectin adhesion between the cells. After 10 min of treatment, the staining became lighter, the cellulose and pectin decreased, and the density decreased. Although the cell structure remained intact and orderly, the cell wall was gradually destroyed. After 20 min of treatment, the cell wall and cell membrane partially or completely disappeared, similar cells began to fuse, the integrity of intercellular substances gradually lost, the phenomenon of inhomogeneity appeared, and the cells began to deform and lose their original shape. After 30 min of treatment, the intracellular substance leaked and holes appeared in the cellulose and pectin between the cells. After 40 min of treatment, the intercellular substance completely disappeared and the cells disintegrated completely or partially. However, the structure and unit of the cell could be distinguished. The citrus mesocarp became thinner and was obviously damaged. After 50 min of treatment, the cell structure completely disappeared, and only the broken fiber fragments remained. In all, these results showed that the crude enzyme produced by ZC-1005 had good degradation effect on the citrus mesocarp by enzymatically hydrolyzing intercellular substances (such as cellulose and pectin) and destroying cell walls and cell membranes.

### Acute Toxicity of Crude Enzymes

To access acute toxicity, the maximum tolerated dose of the crude enzyme was given by intra-gastric gavages about 40 ml/kg of body weight. No abnormal reactions or death was found. In addition, dissection found no obvious gross pathological changes in the treated rats. During the entire observation period, the weight gain percentage of all the treated rats was normal (Table 1). The crude enzyme manifested no obvious acute toxicity under the experimental conditions, indicating that the MTD of the crude enzyme might be higher than 140 ml/kg body weight per day.

**TABLE 1:** Summary of acute toxicity study results for ZC-1005 crude enzymes.

**Gender**	**Group**	**BW**					**Macroscopic observations at necropsy (test)**
		**D0**	**D4**	**D7**	**D10**	**D14**	
Males	Control Test	18.63 ± 0.62^a^	25.62 ± 1.61^a^	26.60 ± 1.46^a^	27.75 ± 1.80^a^	29.10 ± 1.78^a^	NTF
Females	Control Test	18.87 ± 0.56^a^	25.33 ± 1.15^a^	26.69 ± 0.89^a^	27.32 ± 1.21^a^	28.62 ± 1.16^a^	NTF
		19.75 ± 0.89^a^	28.86 ± 1.58^a^	30.93 ± 1.58^a^	33.69 ± 1.82^a^	33.48 ± 1.85^a^	NTF
		19.67 ± 0.42^a^	28.13 ± 1.25^a^	30.39 ± 1.81^a^	33.03 ± 1.67^a^	34.60 ± 1.67^a^	NTF

*BW, body weight (g), mean values of groups of 20 animals.*

*NTF, No treatment-related findings.*

*^*a,b*^In the same column indicate significant differences (*p* < 0.05).*

### Sub-Acute Toxicity

The sub-acute toxicity test was a 28-day study, with the primary outcome of any toxicity caused by long-term feeding. It provides valuable information on the cumulative toxicity of a substance on target organs or the physiological and metabolic effects of long-term exposure to low-dose compounds (OECD 1995). In recent years, it has been widely used to evaluate the safety of enzymes isolated from different microorganisms as food additives and animal feed (Steensma et al., 2004; Akhand et al., 2010; Pariza and Cook, 2010).

#### Evaluation of the Crude Enzymes on Body Weight and Behavior

The observations during the experiment found no abnormal reactions or death. No death occurred during the 28-day test. Compared with the initial value, the body weight of all rats increased significantly (Table 2). However, no significant differences of the weight gain and food conversion efficiency were observed between the control group and the treatment group, which indicated that the crude enzyme had no adverse effects on body weight.

**TABLE 2:** Body weight and food conversion efficiency in SD rats administered for 4 weeks.

**Gender**	**Group**	**Week 0**	**Week 1**		**Week 2**		**Week 3**		**Week 4**	
		**BW**	**BW**	**FCE**	**BW**	**FCE**	**BW**	**FCE**	**BW**	**FCE**
Males	1	122.14 ± 5.40^a^	137.10 ± 5.41^a^	12.61 ± 5.36^a^	180.04 ± 13.97^a^	30.81 ± 4.71^a^	201.60 ± 12.70^a^	21.41 ± 3.65^a^	218.40 ± 12.30^a^	10.93 ± 2.11^a^
	2	123.12 ± 3.43^a^	139.42 ± 4.94^a^	13.33 ± 2.70^a^	183.34 ± 13.28^a^	31.44 ± 5.80^a^	204.56 ± 13.72^a^	20.01 ± 2.48^a^	219.52 ± 13.52^a^	11.42 ± 1.29^a^
Females	1	127.60 ± 3.52^a^	159.40 ± 3.01^a^	21.88 ± 2.79^a^	224.64 ± 5.73^a^	38.28 ± 2.62^a^	289.88 ± 10.34^a^	38.79 ± 4.37^a^	310.66 ± 14.89^a^	12.82 ± 4.33^a^
	2	125.92 ± 7.36^a^	152.74 ± 10.25^a^	19.71 ± 7.00^a^	219.92 ± 7.30^a^	40.63 ± 5.22^a^	278.58 ± 13.56^a^	36.99 ± 7.78^a^	298.46 ± 10.78^a^	12.82 ± 5.69^a^

*Group 1, control; Group 2, 10 ml/kg body weight/day.*

*BW, body weight (g), mean values of groups of 10 animals; FCE, Food conversion efficiency (%) = body weight gain (g)/food consumption (g), mean values of groups of five cages with two animals in each cage.*

*^*a,b*^In the same column indicate significant differences (*p* < 0.05).*

#### Evaluation of Relative Organ Weight by Crude Enzymes

Changes in organ weights are sensitive indicators that reflect the overall health of animals. The relative organ weight of each group is shown in Table 3. Compared with the control group, there was no significant change in organ weight in the treatment group.

**TABLE 3:** The effect of the crude enzymes on organ weights.

**Gender**	**Organs**	**Liver**	**Kidneys**	**Spleen**	**Testicle**
Males	Group 1	9.86 ± 0.19^a^	2.44 ± 0.07^a^	0.71 ± 0.16^a^	3.17 ± 0.24^a^
	Group 2	8.79 ± 0.47^a^	2.26 ± 0.14^a^	0.61 ± 0.09^a^	2.93 ± 0.10^a^
Females	Group 1	6.16 ± 1.13^a^	1.41 ± 0.09^a^	0.40 ± 0.05^a^	–
	Group 2	6.13 ± 0.42^a^	1.44 ± 0.09^a^	0.44 ± 0.04^a^	–

*Values are given as mean ± SD for five rats in each group.*

*Group 1 was untreated and served as the control; Groups 2 was orally administered of the crude enzymes at a dose of 10 ml/kg body weight for 28 days.*

*^*a,b*^In the same column indicate significant differences (*p* < 0.05).*

#### Evaluation of the Crude Enzymes on Hematological and Biochemical Parameters

The hematopoietic system is also one of the most important targets for toxic substances (Ferrario et al., 2008). Several blood parameters were tested to evaluate the changes in the hematopoietic system. The hematological parameters showed no significant difference between control group and the experimental groups (treated with 10 ml/kg BW of the crude enzymes) (Table 4). However, there was a slight increase of platelet concentration in the experimental groups compared with the control group. These findings indicated that the crude enzymes do not induce inflammation in the liver.

**TABLE 4:** The hematological parameters analysis of control group and rats treated with the crude enzymes for 28 days.

**Gender**	**Parameters**	**WBC (10^9^/L)**	**Neu (%)**	**Lym (%)**	**Mon (%)**	**Eos (%)**	**Bas (%)**	**RBC (10^12^/L)**	**HGB (g/L)**	**HCT (%)**	**PLT (10^9^/L)**
Males	Group 1	9.53 ± 2.47^a^	10.26 ± 2.09^a^	86.44 ± 3.73^a^	2.60 ± 1.51^a^	0.60 ± 0.46^a^	0.10 ± 0.00^a^	7.05 ± 0.24^a^	148.40 ± 6.39^a^	41.00 ± 1.85^a^	686.60 ± 63.10^a^
	Group 2	8.56 ± 2.95^a^	10.44 ± 4.00^a^	88.32 ± 3.71^a^	0.96 ± 0.58^a^	0.18 ± 0.15^a^	0.10 ± 0.00^a^	6.99 ± 0.60^a^	150.60 ± 9.81^a^	41.66 ± 2.23^a^	741.80 ± 179.18^a^
Females	Group 1	7.01 ± 1.73^a^	5.50 ± 2.91^a^	93.70 ± 3.38^a^	0.46 ± 0.71^a^	0.24 ± 0.23^a^	0.10 ± 0.00^a^	7.12 ± 0.41^a^	149.80 ± 4.09^a^	41.74 ± 1.25^a^	658.20 ± 46.33^a^
	Group 2	4.93 ± 0.99^a^	9.06 ± 1.79^a^	90.36 ± 1.90^a^	0.28 ± 0.19^a^	0.20 ± 0.10^a^	0.10 ± 0.07^a^	7.02 ± 0.20^a^	151.20 ± 3.49^a^	42.62 ± 1.18^a^	710.40 ± 92.92^a^

*Values are given as mean ± SD for five rats in each group.*

*Group 1 was untreated and served as the control; Groups 2 was orally administered of the crude enzymes at a dose of 10 ml/kg bw for 28 days.*

*^*a,b*^In the same column indicate significant differences (*p* < 0.05).*

AST and ALT, which are biomarkers of hepatocyte lysis in acute liver injury, were measured to evaluate the hepatocyte lysis. There was no significant difference in the activities of AST and ALT between the control and experimental groups, but a slight increase of the AST activity was noted in female rats treated with crude enzyme.

Urea and creatinine, two important markers of renal insufficiency, were measured to evaluate the effect of the crude enzymes on the kidney (Mukinda and Eagles, 2010). There were no differences in serum urea and creatinine levels between the control and experimental groups (Table 5), suggesting that the crude enzymes did not affect the filtration function of the kidney.

**TABLE 5:** Serum biochemical data of control group and rats treated with the crude enzymes for 28 days.

**Parameters**	**Gender**	**Group 1**	**Group 2**
ALB (g/L)	M	31.00 ± 3.00^a^	31.00 ± 1.58^a^
	F	36.40 ± 7.23^a^	32.20 ± 2.77^a^
ALT (U/L)	M	39.40 ± 10.41^a^	40.20 ± 7.16^a^
	F	40.60 ± 9.45^a^	38.00 ± 8.80^a^
AST (U/L)	M	65.80 ± 16.41^a^	73.40 ± 12.64^a^
	F	66.20 ± 15.29^a^	65.00 ± 20.81^a^
BUN (mmol/L)	M	9.35 ± 1.15^a^	8.83 ± 1.04^a^
	F	8.90 ± 1.21^a^	8.17 ± 0.97^a^
CHO (mmol/L)	M	1.77 ± 0.16^a^	1.94 ± 0.38^a^
	F	1.93 ± 0.40^a^	2.17 ± 0.08^a^
Cr (μmol/L)	M	70.80 ± 11.45^a^	72.40 ± 10.01^a^
	F	88.20 ± 8.14^a^	83.80 ± 9.76^a^
GLU1 (mmol/L)	M	7.99 ± 1.50^a^	8.21 ± 0.79^a^
	F	8.29 ± 0.36^a^	6.93 ± 1.20^a^
TG (mmol/L)	M	0.69 ± 0.18^a^	0.70 ± 0.18^a^
	F	0.65 ± 0.19^a^	0.51 ± 0.11^a^
TP (g/L)	M	56.40 ± 10.55^a^	73.00 ± 10.39^a^
	F	67.00 ± 6.71^a^	74.40 ± 8.26^a^

*Values are given as mean ± SD for five rats in each group.*

*^*a,b*^In the same column indicate significant differences (*p* < 0.05).*

### Bone Marrow Micronucleus Test of the Crude Enzymes

The bone marrow micronucleus test showed that there was no significant difference in the rate of micronucleus cells between the control group and the crude enzyme group (Table 6). The PCE/RBC values of the crude enzyme group were all within the normal range. However, the rate of micronucleus cells in the cyclophosphamide group was significantly higher than that of the crude enzyme group and the control group. Under the experimental conditions, the crude enzymes caused no chromosome damage and no increase in the rate of micronucleus cells (Table 6).

**TABLE 6:** The results of bone marrow micronucleus test.

**Group**	**Dose**	**PCE (*n*)**		**Micronucleus cells (*n*)**		**Rate of Micronucleus cells (‰)**		**PCE/NCE**	
		**Male**	**Female**	**Male**	**Female**	**Male**	**Female**	**Male**	**Female**
Control	0	5,000	5,000	6	6	1.20 ± 0.84	1.20 ± 0.84	1.133 ± 0.018	1.133 ± 0.017
The crude enzymes	MTD	5,000	5,000	7	7	1.40 ± 0.55	1.40 ± 1.34	1.119 ± 0.012	1.134 ± 0.023
The crude enzymes	1/2 MTD	5,000	5,000	6	7	1.20 ± 0.45	1.40 ± 0.55	1.129 ± 0.015	1.133 ± 0.028
The crude enzymes	1/4 MTD	5,000	5,000	6	7	1.20 ± 0.84	1.40 ± 1.14	1.140 ± 0.019	1.123 ± 0.022
CP	40 mg/kg	5,000	5,000	146	150	29.20 ± 1.92**	30.00 ± 3.39**	1.059 ± 0.011**	1.048 ± 0.012**

*CP, cyclophosphamide; PCE, polychromatic erythrocytes; NCE, normochromatic erythrocytes.*

Each value is expressed as mean ± SD (*n* = 3).

**Statistically significantly different when compared with the negative control, *p* < 0.01.

### Sperm Aberration Text of the Crude Enzymes

The rate of sperm aberration showed no difference in the control group and the crude enzyme group (Table 7). However, cyclophosphamide was responsible for the germ cell mutation and the increase of abnormal sperm and the rate of sperm aberration. The results showed that the crude enzymes could not cause sperm deformity under the existing experimental conditions.

**TABLE 7:** The results of sperm aberration test.

**Group**	**Dose**	**Mice (*n*)**	**Observed sperms (*n*)**	**Aberrant sperms (*n*)**	**Rate of aberrant sperms (%)**
Control	0	5	5,000	135	2.72 ± 0.29
The crude enzymes	MTD	5	5,000	139	2.78 ± 0.22
The crude enzymes	1/2 MTD	5	5,000	133	2.66 ± 0.34
The crude enzymes	1/4 MTD	5	5,000	128	2.56 ± 0.30
CP	40 mg/kg	5	5,000	433	8.62 ± 0.83**

*CP, cyclophosphamide; MTD, maximum tolerated dose; MTD, maximum tolerated dose.*

Each value is expressed as mean ± SD (*n* = 3).

**Statistically significant different when compared with the negative control *p* < 0.01.

Bone marrow micronucleus test and sperm aberration test are powerful for the evaluation of the potential genotoxicity of chemicals (Chen et al., 2001; Chandrasekaran et al., 2009). According to the above results, the crude enzymes manifested no significant acute oral toxicity and obvious sub-acute oral toxicity under the experimental conditions.

### Genome Features of ZC-1005

**FIGURE 2:**
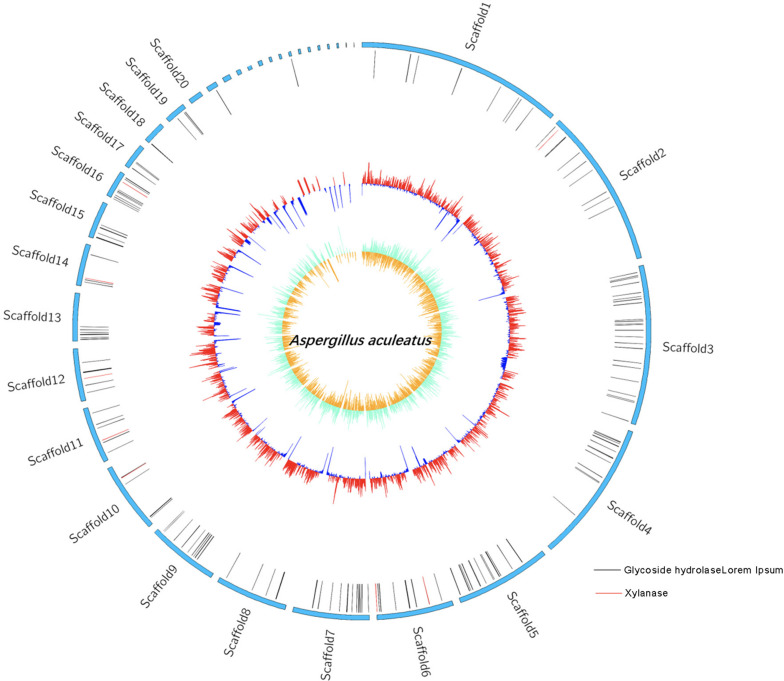
Genome map of the ZC-1005.

The whole genome sequencing of ZC-1005 was carried out to explore the mechanism of the degradation. As shown in Figure 2 and Table 8, ZC-1005 genome was assembled into 35 scaffolds of 35,458,484 bp with a G + C content of 52.53%. A total of 10,147 protein coding genes were predicted, and 358 tRNAs and 94 rRNA operons were identified, namely, 43 5S rRNA genes, 25 16S rRNA genes, and 26 23S rRNA genes. According to the gene prediction, a total of 20,817,415 bp genes were found. The ratio of gene length/genome was 41.29%, and the GC content of genes and intergenetic regions were 55.85 and 47.8%, respectively. In addition, there were 316 interspersed repetitive sequences, including 10 LTR (long terminal repeats), 46 DNA transposons, 157 LINE (long interspersed repeated segments), 102 SINE (short interspersed repeated segments), and one unclassified.

**TABLE 8:** The genome features of ZC-1005.

**Attributes**	**Characteristic**
Genome size (bp)	35,458,484
G + C content (%)	52.53%
GC content in gene region (%)	55.85%
GC content in intergenetic region (%)	47.8%
Protein-coding genes (CDS)	10,147
Gene total len (bp)	20,817,415
Gene/genome (%)	58.71%
Intergenetic region len (bp)	14,642,069
Intergenetic len/genome (%)	41.29%
tRNA genes	358
5S rRNA	43
16S rRNA	25
23S rRNA	26
Genes assigned to NR	10,146
Genes assigned to Swiss-Prot	7,003
Genes assigned to Pfam	7,499
Genes assigned to COG	8,550
Genes assigned to GO	6,070
Genes assigned to KEGG	3,313
Genes assigned to CAZy	604

### Gene Function Analysis

Our previous study showed that the crude enzyme solution of ZC-1005 had high xylanase activity (Zhang, 2014), so in this study, we hope to find specific genes related to xylanase production through whole genome sequencing and genome annotation analysis. Non-Redundant Protein Database (NR) annotation displays the annotation information of each gene in each sample, including function description and comparison (Deng et al., 2006). Gene 01512 functioned as xylanolytic transcriptional activator XlnR; gene 05833 could encode endo-1,4-β-xylanase B precursor; gene 05469 was related to xylanase GH10; gene 07781 was one type of polysaccharide deacetylase gene; gene 08432 was described as an endo-1,4-β-xylanase A and endo-1,4-β-xylanase B precursor; both gene 09042 and gene 09694 could encode endo-1,4-β-xylanase; gene 08008 could assist the production of xylanolytic transcriptional activator and xylanolytic transcriptional activator XlnR. All these annotations above provided basic information for further research and practical application in the future, such as cloning and expression of carboxymethyl cellulase and xylanase, and enzyme structure research.

#### COG Analysis

**FIGURE 3:**
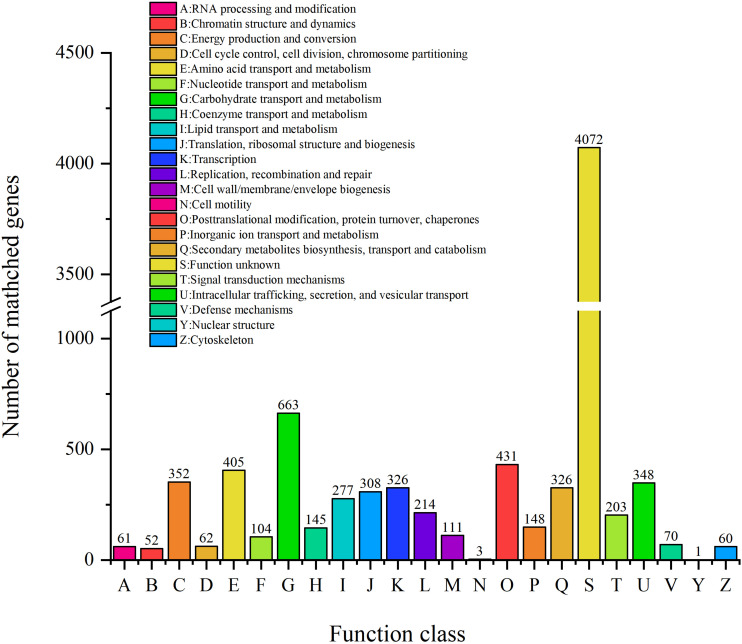
The COG function classification of ZC-1005 genome.

Clusters of orthologous groups analysis was performed to understand how ZC-1005 deployed genes in its genome (Tatusov et al., 2003). As shown in Figure 3, a total of 8,550 genes were categorized into 23 functional types. The major categories were carbohydrate transport and metabolism (G); posttranslational modification, protein turnover, chaperones (O); amino acid transport and metabolism (E); energy production and conversion (C); and intracellular trafficking, secretion, and vesicular transport (U).

To elucidate the role of ZC-1005 in cellulose degradation at the genetic level, we analyzed specific COGs involved in carbohydrate metabolism. A total of 663 genes have been annotated into carbohydrate metabolism. The most abundant COGs were COG0477 (major facilitator superfamily), COG1472 (hydrolase family 3), COG3325 (chitinase), COG0366 (α-amylase), COG2273 (hydrolase family 16), COG1501 (hydrolase, family 31), and COG2730 (glycoside hydrolase family 5). COG0477 is a secondary active transporter that catalyzes the transport of various substrates (Madej et al., 2014). It couples the movement of the substrates with the proton motive force generated across the cell membrane, instead of combining with ATP hydrolysis (Nitika et al., 2016). COG0366 encodes an α-amylase that hydrolyzes large alpha-linked polysaccharides, such as starch and glycogen, to produce glucose and maltose (Štefan and Gabriško, 2016).

#### GO Term Annotations

**FIGURE 4:**
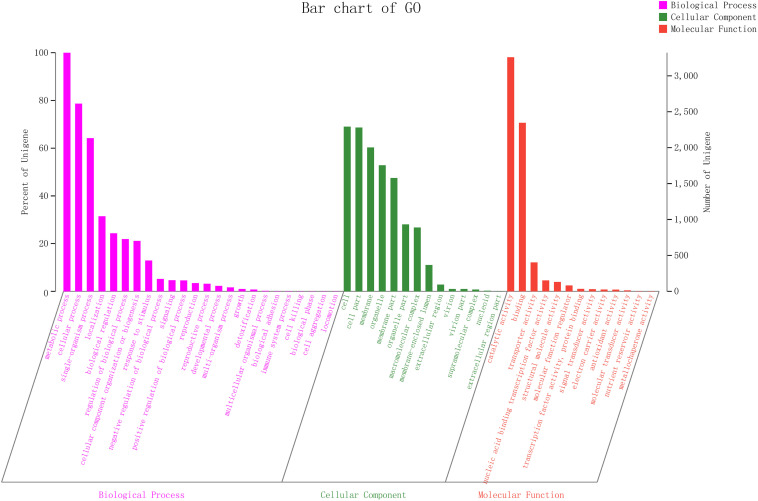
Gene ontology (GO) slim classifications for the ZC-1005.

To explain the biological correlation of the genome of ZC-1005, the genes were classified into three categories by GO analysis based on the degree of matching with the known sequences. The numbers of GO items and genes in the three categories were as follows: molecular function (gene number: 4,582), biological process (gene number: 4,437), and cellular component (gene number: 3,795) (Figure 4). Genes in the biological process category were divided into 24 sub-functions, among which metabolic process (GO: 0008152, gene number 3,312), cellular process (GO: 0009987, 2,613), and single-organism process (GO: 0044699, 2,132) were the most abundant; the cellular component category contained 14 sub-functions of genes, most of which were related to functions of the cell (GO: 0005623, 2,292), cell part (GO: 0044464, 2,279), and membrane (GO: 0016020, 2,002); the molecular function category had 13 sub-functions of genes, most of which involved catalytic activity (GO: 0003824, 3,258) and binding (GO: 0005488, 2,346). According to the GO annotation of ZC-1005, genes in the metabolic process (GO: 0008152), catalytic activity (GO: 0003824), and cellular process (GO: 0009987) sub-functions were the three most abundant.

#### KEGG Annotations

**FIGURE 5:**
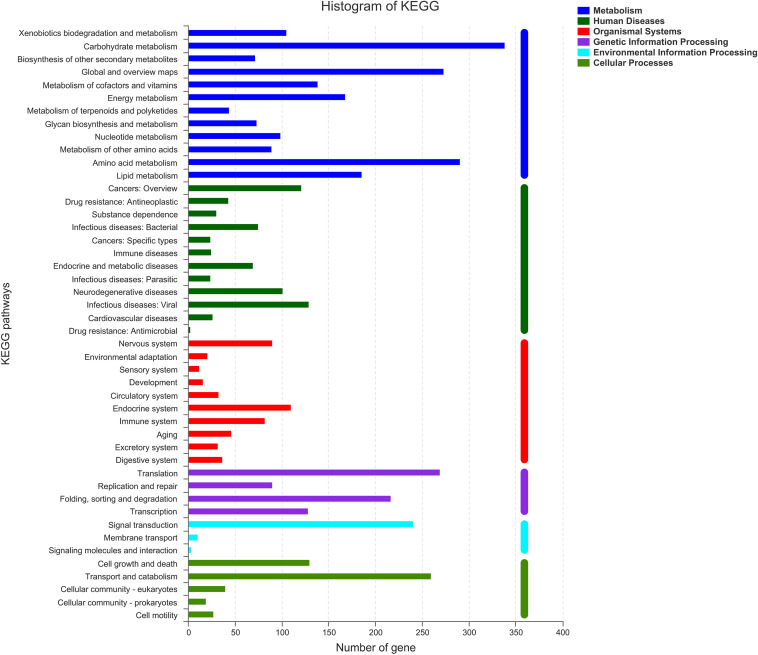
Clusters of KEGG annotation.

As shown in Figure 5, there were six classifications of KEGG pathways: the metabolic genes were the most abundant, followed by human diseases. In KEGG metabolism annotations, carbohydrate metabolism and amino acid metabolism were the main functions, with 339 and 291 genes, respectively. In these metabolic processes, pathways such as amino and nucleotide sugar metabolism (ko00520), glycolysis/gluconeogenesis (ko00010), and starch and sucrose metabolism (ko00500) were dominant. Fifty-eight genes were found to be associated with ko00520.

#### Carbohydrate-Active Enzyme Annotation

**FIGURE 6:**
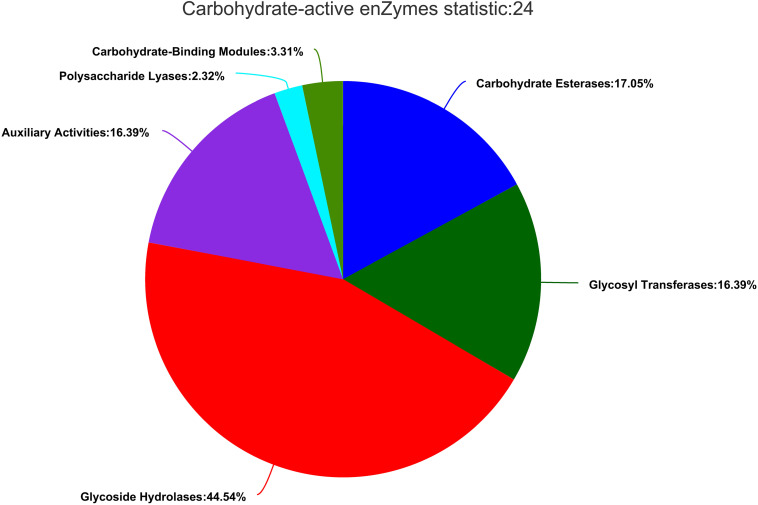
Gene count distributions of carbohydrate-active enzyme (CAZy) families.

Carbohydrate-active enzymes (CAZymes) fell into different categories and families in the CAZy database^2^ (Coutinho and Henrissat, 1999). The results showed that there were 604 genes in CAZy families falling into five subfamilies. In ZC-1005, glycoside hydrolases (GHs) accounted for a large proportion, and the genes involved in carbohydrates degradation account for 44.54% (269). Additionally, carbohydrate esterase (CEs, 103, 17.05%), glycosyl transferases (GTs, 99, 16.39%), polysaccharide lyases (PLs, 14, 2.32%), enzymes with auxiliary activities (AAs, 99, 16.39%), and carbohydrate-binding modules (CBMs, 20, 3.31%) were identified (Figure 6). The GHs and GTs family had the highest number of enzyme genes and played a key role in the cleavage of polymer substrates (Roth et al., 2017).

Hemicellulase mainly includes xylanase (EC3.2.1.8), β-galactosidase (EC 3.2.1.23), β-mannosidase (EC3.2.1.25), β-glucuronidase (EC 3.2.1.31), β-xylosidase (EC 3.2.1.37), β-D-fucosidase (EC 3.2.1.38), and α-L-arabinofuranosidase (EC 3.2.1.55). Ten GH76, seven GH31, four GH2, and four GH92, which were related to the degradation of mannose, were annotated in the CAZyme annotation. Meanwhile, 13 GH43, 3 GH11, 2 GH10, 2 GH51, and 2 GH74 relevant to xylan degradation were also annotated, while cellulase mainly includes endoglucanase (EC3.2.1.4), β-glucosidase (EC 3.2.1.21), and exoglucanase (EC 3.2.1.91). From the CAZyme annotation, several interesting genes related to cellulose degradation were screened out. For example, 4 GH55, 4 GH12, 2 GH7, and 1 GH6 were related to endoglucanase, and 18 GH3 and 3 GH1 were relevant to β-glucosidase.

## Discussion

In recent years, more than 200 microorganisms that can degrade cellulose and hemicellulose were found, mainly derived from compost, rumen, anaerobic sludge, and soil bacteria and fungi (Tsapekos et al., 2017). Filamentous fungi are the main microbial strains for cellulase production, including *Trichoderma*, *Aspergillus*, and *Penicillium* (Zhang et al., 2003). These studies mainly focused on the production process of xylanase (such as culture conditions and enzyme inducers) (Ramanjaneyulu and Rajasekhar Reddy, 2016; Sunkar et al., 2020), purification and identification of xylanase, and enzymatic characteristics (Gao et al., 2017; Sherien et al., 2020; Torkashvand et al., 2020; Cao et al., 2021). *Aspergillus niger* (Diogo et al., 2018), *Trichoderma* (Schmoll, 2018), *Streptomyces cirratus* (Zhang, 2004), *Streptomyces lividans* (Dupont et al., 1996), *Streptomyces olivochromogenes* (Gregory et al., 1987), and rumen microorganisms (Yousuf et al., 2010) are the main microorganisms that degrade hemicellulose. These enzymes have different structures and ways of action. However, the principal degradation mechanisms are almost the same: destroy the chemical bond and cleave the lignocellulose structure (Cragg et al., 2015; Wang et al., 2017).

Both fungi and bacteria are able to degrade cellulose, but with different characters. For bacteria, *Cytophaga* (Zhu et al., 2016), *Cellulomonas*, *Acidothermus cellulolyticus*, *Ncardiac*, and *Streptomyces* (Liu et al., 2007) are the main species. Most aerobic bacteria secrete single-cellulose extracellular enzymes, which shows low degradation efficiency and poor practicability (Jrgensen and Pinelo, 2016). Anaerobic bacteria have the advantages of high degradation efficiency and strong resistance to contamination caused by miscellaneous bacteria, but the growth rate is obviously low. Moreover, the growth rate would be probably inhibited by degradation intermediates such as pentose and formic acid (Qing et al., 2010). However, for fungi, the cellulase system is a whole enzyme system, with high degradation activity. *Trichoderma* (Karlsson et al., 2002), *Rhizopus*, *Aspergillus* (Ahmad and Khare, 2018), and *Penicillium* (Jian et al., 2018) are widely used in cellulase production, among which *Trichoderma reesei* and *Trichoderma viride* are the mostly used (Wen et al., 2010).

Hemicellulase secreted by microorganism fermentation (mainly xylanase and mannanase) often has high enzyme activity (Bradner et al., 2010), but its application in the food industry is limited due to the potential food safety hazards. Thus, in this study, the biological safety was evaluated to ensure the safety of the crude enzyme for its use in the food industry. Such safety evaluations were also studied by other research groups. Sewalt et al. (2018) investigated the safety of a synthetic variant *Cytophaga* sp. α-amylase enzyme expressed in *Bacillus licheniformis* and found that the α-amylase is safe for both human and animal food. Silano et al. (2018) have studied the safety evaluation of the food enzyme xylanase from a *Bacillus subtilis*; their results indicated that the enzyme showed no toxicity. These results were consistent with ours. Safety evaluation of microbial enzymes is an essential procedure before their use in the food industry; such evaluations are required not only for registration purposes but also for health purposes.

From the results of genome analysis and annotation, many interesting findings were screened out for estimating the mechanisms of degradation. Firstly, according to the results of KEGG, xylanase together with β-D-xylosidase 4 [EC: 3.2.1.37] present in ko00520 were involved in the hydrolysis of xyloside. In addition, xylooligosaccharide could be hydrolyzed into xylose by excision of β-D-xylosidase 4 from the non-reducing end (Wan, 2016). In the genome, 51 genes were found in ko00010, in which D-glucose was phosphorylated to D-glucose-6-phosphate. Also, ko00010 was related to other pathways. For example, D-glucose-6-phosphate could be converted to pyruvate, and pyruvate could be oxidized to acetyl-CoA that was able to enter the citrate cycle. There were 45 genes associated with ko00500, and enzyme endoglucanase (EC.3.2.1.4), which was common in ko00500, was involved in cellulose degradation. There were starch and sucrose metabolic pathways in ZC-1005, which indicated that cellulose could be hydrolyzed into cellobiose and finally to β-D-glucose. Secondly, according to the results of CAZyme annotation, many GH families related to cellulose degradation were screened out. The GH family is a ubiquitous group of enzymes that act on the glycosidic bond. For example, β-glucosidase [cellulase, EC 3.2.1.21] produced by ZC-1005 with cellulose degradation function usually belongs to the GH3 (Suthangkornkul et al., 2016) and GH5 families (Pandey et al., 2014). In the genome, there were 15 GH13s hydrolyzed starch, such as α-glucosidase [amylase, Family: GH13, EC 3.2.1.20], α-amylase [amylase, EC 3.2.1.1], and α-glycosidase [amylase, EC 3.2.1] (Natália et al., 2016). Due to the action of enzymes, four GH12, three GH1, two GH7, and one GH6 showed potential cellulose degradation ability. Additionally, seven GH31, four GH92, and four GH2 were responsible for mannose degradation (Bohra et al., 2018). Three GH11, 2 GH10, and 13 GH43 were important components of the xylan degradation system (Romero et al., 2012); they were considered important members of hemicellulose degradation. Meanwhile, many CEs involved in the xylan degradation were also found in the genome, including 61 CE10s, 12 CE1s, 6 CE4s, 6 CE5s, 3 CE12s and 1 CE2s. The CE1, CE4, CE5, CE12, and CE2 family all possess acetyl xylan esterase [xylanase, EC 3.1.1.6], which could enhance xylan solubilization (Zhang, 2011). CE10 showed carboxylesterase [hemicellulase, EC 3.1.1.1] and xylanase [hemicellulase, EC 3.2.1.8] activities related to hemicellulose degradation (Zhao et al., 2014). Polysaccharide deacetylases played a key role in degrading polysaccharides. CE4 contained highly specific acetyl xylan esterases [xylanase, EC 3.1.1.72] and peptidoglycan N-deacetylates, which were involved in the degradation of chitin (Biely, 2012). Moreover, six PL1s and four PL4s were annotated to degrade pectin (Chen et al., 2013). Pectate lyase (pectinase, EC 4.2.2.2) can usually cleave (1→4)-α-d-galacturonan, resulting in the presence of oligosaccharides at the end (See-Too et al., 2017). Moreover, the CAZy auxiliary activity family 3 (AA3) contains enzymes from the glucose–methanol–choline (GMC) family of oxidoreductases. These enzymes can enhance the activity of other AA family enzymes *via* their reaction products or support the role of glycoside hydrolases in the degradation of lignocellulose (Sützl et al., 2018). AA4 contained vanillyl-alcohol oxidases, which could convert some phenols (Heuvel et al., 2002). AA7 enzymes participated in the biotransformation or detoxification of lignocelluloses (Levasseur et al., 2013). Additionally, the AA9 (formerly GH61) protein is a copper-dependent lytic polysaccharide monooxygenase (LPMO), and it has been reported many times that cellulose chains cleaved with the oxidation of various carbons (C-1, C-4, and C-6). In all, the high diversity of functional annotations indicated that ZC-1005 had a strong ability to degrade hemicellulose. All the findings mentioned above may partially explain the mechanism for the degradation of the crude enzyme from ZC-1005.

## Conclusion

In conclusion, the results showed that the crude enzymes of ZC-1005 were capable to degrade citrus mesocarp. From the whole genome analysis of ZC-1005, we predicted several genes associated with xylanase; the high percentage of carbohydrate transport and metabolism (7.58%) and amino acid transport and metabolism (4.63%) functions indicated that ZC-1005 had a great potential to degrade proteins and carbohydrates. Further studies on the mechanism of ZC-1005 showed that the strain had different diversity groups of glycosyl hydrolase (GH) family genes, which are vital to cellulolytic and hemicellulolytic biomass degradation. Meanwhile, we also validated the high biological safety for the crude enzymes of ZC-1005; together with its good degradation ability, ZC-1005 and the crude enzyme secreted from the strain are expected to be applied in citrus processing industries as a potential degradation solution. In the future, based on the genes screened out in our study, more transgenic studies will be carried out to conduct an expression system in procaryotic and/or eukaryotic organisms. Our results showed that the citrus enzymatic decapsulation technology used in our study was efficacious and safe for canned citrus product processing, which may also solve the industrial waste problem. Therefore, ZC-1005 and the crude enzyme secreted from the strain were very promising to be used in the citrus processing industry.

## Data Availability Statement

The datasets presented in this study can be found in online repositories. The names of the repository/repositories and accession number(s) can be found below: https://www.ncbi.nlm.nih.gov/bioproject/PRJNA695560.

## Ethics Statement

The animal study was reviewed and approved by Institutional Animal Care and Use Committee (IACUC) of Hunan Sileike Jingda Co., with certificate no. IACUC-SJA18072.

## Author Contributions

JG: conceptualization. JW: methodology and software. JW, YQ, and XH: validation. JW and ZG: writing—original draft preparation and visualization. JG and ZG: writing—review and editing. JG, GL, FF, and YS: supervision. YS: project administration and funding acquisition. All authors contributed to the article and approved the submitted version.

## Conflict of Interest

The authors declare that the research was conducted in the absence of any commercial or financial relationships that could be construed as a potential conflict of interest.

## Publisher’s Note

All claims expressed in this article are solely those of the authors and do not necessarily represent those of their affiliated organizations, or those of the publisher, the editors and the reviewers. Any product that may be evaluated in this article, or claim that may be made by its manufacturer, is not guaranteed or endorsed by the publisher.

## Footnotes

1
www.i-sanger.com

2
http://www.cazy.org
